# Vibration acceleration enhances proliferation, migration, and maturation of C2C12 cells and promotes regeneration of muscle injury in male rats

**DOI:** 10.14814/phy2.15905

**Published:** 2024-02-23

**Authors:** Seira Sato, Tatsuhiro Hanai, Takashi Kanamoto, Fuminori Kawano, Minami Hikida, Hiroyuki Yokoi, Yasuhiro Take, Takuya Magome, Kosuke Ebina, Tatsuo Mae, Hiroyuki Tanaka, Ken Nakata

**Affiliations:** ^1^ Department of Sports Medical Science Osaka University Graduate School of Medicine Suita Osaka Japan; ^2^ Department of Medicine for Sports and Performing Arts Osaka University Graduate School of Medicine Suita Osaka Japan; ^3^ Graduate School of Health Sciences Matsumoto University Matsumoto Nagano Japan; ^4^ Department of Oral and Maxillofacial Surgery Nihon University School of Dentistry Chiyoda‐ku Tokyo Japan; ^5^ Yokoi Health Care and Sports Orthopaedics Clinic Toyonaka Osaka Japan; ^6^ Department of Musculoskeletal Regenerative Medicine Osaka University Graduate School of Medicine Suita Osaka Japan; ^7^ Department of Sports Medical Biomechanics Osaka University Graduate School of Medicine Suita Osaka Japan

**Keywords:** C2C12 cells, muscle regeneration, rats, vibration acceleration, whole‐body vibration

## Abstract

Vibration acceleration (VA) using a whole‐body vibration device is beneficial for skeletal muscles. However, its effect at the cellular level remains unclear. We aimed to investigate the effects of VA on muscles in vitro and in vivo using the C2C12 mouse myoblast cell line and cardiotoxin‐induced injury in male rat soleus muscles. Cell proliferation was evaluated using the WST/CCK‐8 assay and proportion of Ki‐67 positive cells. Cell migration was assessed using wound‐healing assay. Cell differentiation was examined by the maturation index in immunostained cultured myotubes and real‐time polymerase chain reaction. Regeneration of soleus muscle in rats was assessed by recruitment of satellite cells, cross‐sectional area of regenerated muscle fibers, number of centrally nucleated fibers, and conversion of regenerated muscle from fast‐ to slow‐twitch. VA at 30 Hz with low amplitude for 10 min promoted C2C12 cell proliferation, migration, and myotube maturation, without promoting expression of genes related to differentiation. VA significantly increased Pax7‐stained satellite cells and centrally nucleated fibers in injured soleus muscles on Day 7 and promoted conversion of fast‐ to slow‐twitch muscle fibers with an increase in the mean cross‐sectional area of regenerated muscle fibers on Day 14. VA enhanced the proliferation, migration, and maturation of C2C12 myoblasts and regeneration of injured rat muscles.

## 1 INTRODUCTION

Vibration acceleration (VA), using whole‐body vibration (WBV) devices, is being increasingly utilized during exercise and rehabilitation (Stania et al., 2016). VA using a Power Plate® (Performance Health Systems, LLC, Northbrook, IL) promoted rib fracture healing in rats (Uchida et al., 2017) and could promote chondrogenic differentiation of ATDC5 and C3H10T1/2 cells in vitro (Yokoi et al., 2020). These results suggest that VA directly could affect cells and promote fracture healing.

Cells sense mechanical stresses such as strain, compressive pressure, fluid flow, pulse, and vibration, and convert these stimuli into biochemical signals that affect cellular morphology, proliferation, differentiation, and motility (Batra et al., 2012; Chen et al., 2013; Klein‐Nulend et al., 1997). Skeletal muscles are highly responsive to mechanical stress. In addition, various types of mechanical stimulations, such as stretching (Chen et al., 2013) and ultrasound (Chan et al., 2010), effectively promote muscle regeneration and satellite cell proliferation. Applying the proper amount of mechanical stress is important to control the cell's ability to proliferate and differentiate, thereby maintaining homeostasis and organ function, as a lack of mechanical stress leads to muscle loss (Gao et al., 2018), whereas excess stress leads to muscle injury (Wilder & Sethi, 2004).

Recently, WBV has been reported to have positive effects on muscle strength (Delecluse et al., 2003), flexibility (Fowler et al., 2019), muscular performance (Torvinen et al., 2002), and delayed‐onset muscle soreness (Rhea et al., 2009) in humans. Furthermore, it has been reported that WBV promotes muscle repair in a mouse model of laceration injury (Corbiere et al., 2018). Although few reports have examined the effects of VA both in vitro and in vivo, whether VA could directly affect muscle cells remains unclear.

This study aimed to investigate the effects of VA on muscles in vitro and in vivo using the mouse myoblast cell line, C2C12 and a rat model of cardiotoxin‐induced muscle injury. We hypothesized that VA could have positive effects on various biological events, such as the proliferation, migration, and differentiation of C2C12 cells in vitro, and promote muscle regeneration in vivo.

## 2 MATERIALS AND METHODS

### 2.1 Vibration acceleration

A Power Plate® Pro5 HPTM was used to apply VA. This machine provided mechanical loading of alternative gravitational acceleration using three‐dimensional vibration at a selected frequency (30 or 50 Hz) with low (approximately 2.5 mm) or high (approximately 5.0 mm) amplitude. If the gravitational acceleration (1 G) was set as baseline, with the direction of gravity being positive, acceleration conditions of 30 Hz/low amplitude (30‐Low), 30 Hz/high amplitude (30‐High), 50 Hz/low amplitude (50‐Low), and 50 Hz/high amplitude (50‐High) could achieve peak magnitudes of roughly 1.5 G, 3 G, 3 G, and 6 G, respectively. As previously described (Uchida et al., 2017; Yokoi et al., 2020), all experiments were performed with VA stimulation at 30‐Low for 10 min, except for the investigation of the differences caused by VA intensity on C2C12 proliferation.

### 2.2 Cell culture experiments

**FIGURE 1:**
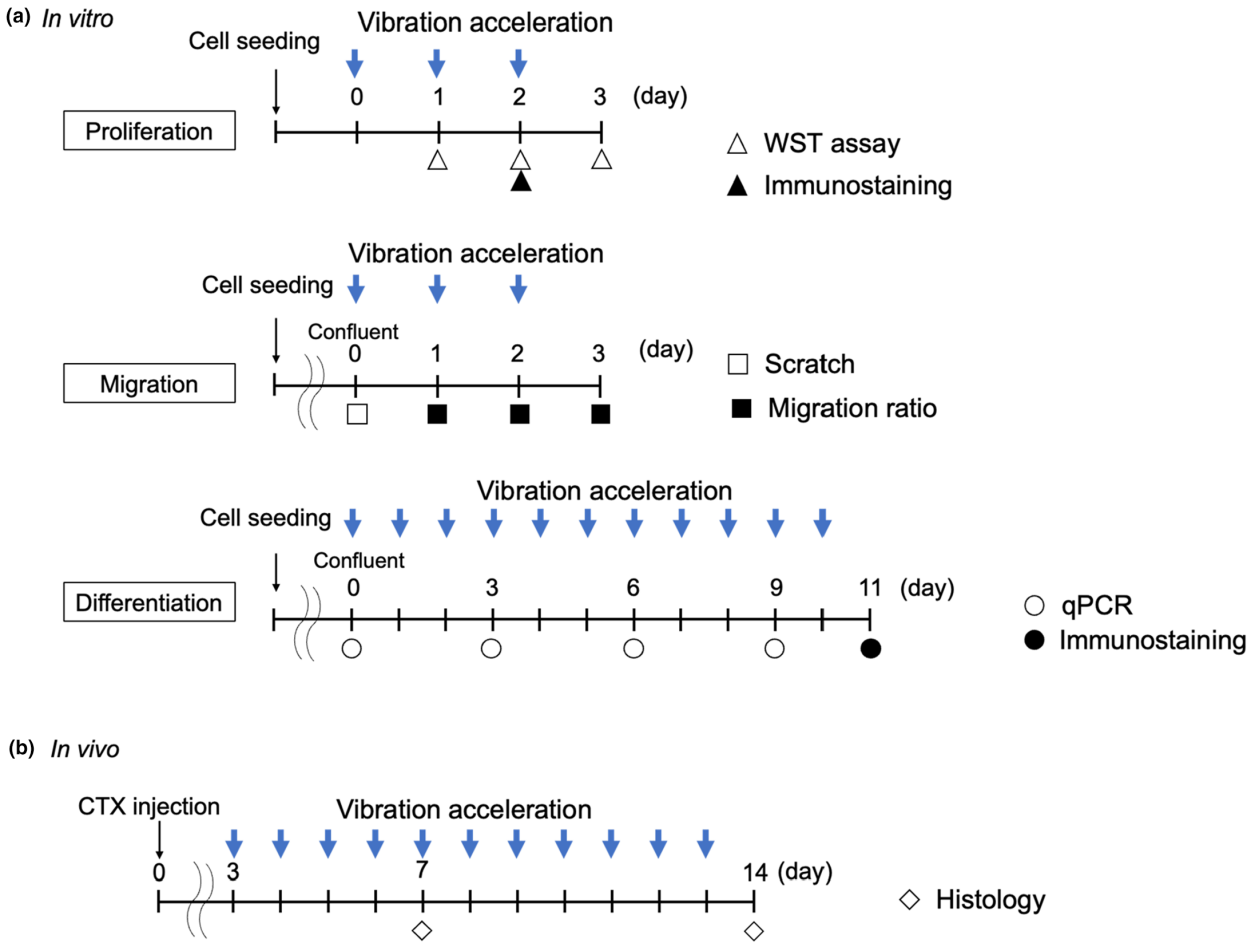
Study design to examine the effects of vibration acceleration on muscles in vitro and in vivo using C2C12 cells (a) and a rat model of cardiotoxin‐induced muscle injury (b).

C2C12, ATDC5, C3H10T1/2, and MC3T3‐E1 cells were obtained from the Riken BioResource Research Center (Ibaraki, Japan) and cultured under 5% CO_2_ at 37°C. The cells were seeded at density of 5 × 10^3^ cells/cm^2^. C2C12, C3H10T1/2, and HeLa cells were cultured in low glucose Dulbecco's modified Eagle's medium (DMEM) (#D6046; Sigma‐Aldrich, MO, USA) supplemented with 10% fetal bovine serum (FBS; #175012; Nichirei Biosciences Inc. Tokyo, Japan), and 1% penicillin/streptomycin (P/S; #15140122; Thermo Fisher Scientific, MA, USA). ATDC5 cells were maintained in DMEM/Ham's F‐12 (#D8062; Sigma‐Aldrich) with 10% FBS and 1% P/S, whereas MC3T3‐E1 cells were maintained in α‐MEM (#C12571500BT; Thermo Fisher Scientific) with 10% FBS and 1% P/S. The medium was changed every day. To examine the differentiation of C2C12 cells, the culture medium was replaced with medium containing 2% horse serum (HS; #16050130; Thermo Fisher Scientific) and 1% P/S after reaching confluency. In the control group, cells were cultured statically, and in the VA group, culture plates/dishes were placed on Power Plate® and stimulated once daily for 10 or 30 min. During stimulation, the control dishes were placed outside the incubator to create an environment similar to that of the VA group. The first day of VA was set as Day 0. To examine cell proliferation, cells were subjected to VA on the day after cell seeding. Moreover, to examine cell differentiation, cells were subjected to VA after being transferred to differentiation medium (Figure 1a).

### 2.3 Cell proliferation assay

To assess cell viability, the WST/CCK‐8 assay (#343–07623; Dojindo, Kumamoto, Japan) was performed every 24 h after VA according to the manufacturer's instructions. Cells were cultured in 24‐well plates for up to 3 days with or without VA. After incubation for 1 h at 37°C, the optical density (OD) at 450 nm was measured using a microplate reader. Cell viability ratio was calculated compared to the OD in the control group on Day 1.

### 2.4 Immunocytochemistry

C2C12 cells were cultured in a 35 mm dish and immunostained with Ki67, a marker for cell proliferation, 2 days after the first VA stimulation, and with myosin heavy chain (MHC) on Day 11 for evaluating myotube formation in differentiated C2C12 cells. The cells were washed with phosphate buffered saline (PBS), fixed in 4% paraformaldehyde/PBS for 20 min, permeabilized with 0.1% Triton X‐100 in PBS for 15 min, and blocked with 2% bovine serum albumin (BSA; #01862–87; Nakalai Tesque Inc., Kyoto, Japan)/PBS for 30 min at room temperature. The cells were then incubated with primary antibodies for 2 h at room temperature, followed by fluorescence‐conjugated secondary antibodies for 1 h at room temperature and counterstained with 4′,6‐diamidino‐2‐phenylin‐dole (DAPI; #H‐1200; Vector Laboratories Inc., CA, USA) for nuclei. The primary and secondary antibodies used were rabbit monoclonal anti‐Ki67 (#9129; Cell Signaling Technology, Danvers, MA, USA), mouse monoclonal anti‐slow skeletal MHC (#ab11083; Abcam plc., Cambridge, UK), donkey anti‐rabbit Alexa Fluor 488 (#A‐21206; Thermo Fisher Scientific), and goat anti‐mouse Alexa Fluor 555 (#A‐21137; Thermo Fisher Scientific). Fluorescent signals were imaged by a Leica DMi8 inverted microscope (Leica Microsystems, Wetzlar, Germany) equipped with Leica DFC9000 GT sCMOS camera (Leica Microsystems) and Leica HC PL FLUOTAR objective (Leica Microsystems). The captured pictures were processed using LAS X software. Fields (magnification ×200) of view were randomly selected for each experiment. The proportion of Ki67‐positive cells (%) was calculated using ImageJ software as the number of cells stained with Ki67 divided by the number of cells stained with DAPI. To evaluate the maturation of C2C12 myotubes, the maturation index was calculated by the number of fibers in the MHC‐stained region with more than five nuclei divided by the total number of myotubes, as previously described (Ribeiro et al., 2018); the index of the VA group was calculated relative to the control group, with a value of 1.

### 2.5 Wound healing assay

An in vitro wound‐healing assay was performed by mechanically disrupting confluent monolayers of C2C12 cells, as previously described (Okamoto et al., 2014). The cells were divided into two groups: VA and control. Wound distance between scratch edges was measured every 24 h in the same region of the plate. Migration ratio was calculated by dividing the wound distance by the initial distance.

### 2.6 Quantitative real‐time polymerase chain reaction (qRT‐PCR)

Total RNA was extracted from C2C12 cells using PureLink RNA Mini Kit (#12183018A; Thermo Fisher Scientific) according to the manufacturer's protocol. Complementary DNA (cDNA) was obtained using a high‐capacity RNA‐to‐cDNA kit (#4387406; Thermo Fisher Scientific), and qRT‐PCR was performed using SYBR Green (#4367659; Thermo Fisher Scientific). The expression levels of myogenic genes (*MyoD*, *Myf5*, *Myogenin*, *Myomaker*, and *Myomixer*) were determined using sequence‐specific primers (Table 1; 10 μM; Integrate DNA Technologies, Inc., Coralville, IA, USA) and examined on Days 0, 3, 6, and 9. Gene expression of glyceraldehyde 3‐phosphate dehydrogenase (*GAPDH*) was used as the internal reference. The relative expression level of each target gene was normalized to the internal standard control using the 2^−ΔΔCT^ method.

**TABLE 1:** Sequence specific primers.

Gene	Forward primer (5′–3′)	Reverse primer (5′–3′)
*GAPDH*	GGCAAATTCAACGGCACAGTC	AAGCAGTTGGTGGTGCAGGA
*MyoD*	CCACTCCGGGACATAGACTTG	AAAAGCGCAGGTCTGGTGAG
*Myf5*	ACAGCAGCTTTGACAGCATC	AAGCAATCCAAGCTGGACAC
*Myogenin*	GAGACATCCCCCTATTTCTACCA	GCTCAGTCCGCTCATAGCC
*Myomaker*	ATCGCTACCAAGAGGCGTT	CACAGCACAGACAAACCAGG
*Myomixer*	GTTAGAACTGGTGAGCAGGAG	CCATCGGGAGCAATGGAA

Abbreviation: GAPDH, glyceraldehyde 3‐phosphate dehydrogenase.

### 2.7 In vivo animal experiments

All animal experiments were approved by the Ethics Review Committee for Animal Experimentation of the Graduate School of Medicine, Osaka University (approval number: 27–095‐000). Twenty‐four 7‐week‐old male Wistar Hannover rats were obtained from CLEA Japan Inc. (Tokyo, Japan) and housed in constant temperature with a 12:12‐h light–dark cycle and ad libitum access to food (Rodent Diet CE‐2; CLEA Japan) and water. At 8 weeks of age, the rats were deeply anesthetized by administration of 5% isoflurane via inhalation and pentobarbital via subcutaneous injection (50 mg/kg). A skin incision was made on the lateral right lower leg of rat to expose the space between the gastrocnemius and soleus muscles. Thereafter, 0.1mL of cardiotoxin (0.03% cardiotoxin/PBS, # L8102‐1MG; Latoxan, Rosans, France), a protein kinase C‐specific inhibitor, was injected under a magnifying glass into the center of the soleus muscle belly to induce skeletal muscle injury. The left soleus was not subjected to any treatment. After awakening from anesthesia, the animals were allowed to move freely in their cages. The rats were divided into the VA and control groups, 12 each. Although rats in the VA group were subjected to VA at 30‐Low for 10 min, rats in the control group were placed on the Power Plate® to maintain comparable conditions without VA. VA was started 3 days after soleus muscle injury and was applied daily at 10:00 a.m. Seven and 14 days after the injury, six rats each in the VA and control groups were euthanized (Figure 1b).

### 2.8 Histology of the soleus muscle in rats

The injured soleus muscle was harvested, frozen in liquid nitrogen, and stored at −80°C until the analyses. The center of fresh‐frozen soleus muscles was transversely sectioned and 10‐μm axial sections were processed for hematoxylin–eosin (H&E) staining and immunohistochemistry examination. For immunofluorescence, the muscle sections were fixed in 4% paraformaldehyde/PBS for 5 min and −20°C methanol for 10 min, followed by blocking with 1% donkey serum (#ab7475; Abcam) for 20 min. The tissue sections were incubated with the primary antibodies, mouse monoclonal anti‐Pax7 antibody (#MAB1675; R&D systems; Minneapolis, MN), mouse monoclonal anti‐dystrophin antibody (#MA5‐49307; Thermo Fisher Scientific), rabbit polyclonal anti‐laminin antibody (#ab11575; Abcam), rabbit polyclonal anti‐desmin antibody (#PA5‐85187; Thermo Fisher Scientific), rabbit polyclonal anti‐type‐I MHC antibody (#MA5‐28196; Thermo Fisher Scientific), and rabbit polyclonal anti‐type‐II MHC antibody (#MA1‐90701; Thermo Fisher Scientific) for overnight at 4°C. Subsequently, the sections were reacted with secondary antibodies, donkey anti‐rabbit Alexa Fluor 488 or donkey anti‐mouse Alexa Fluor 594 (#A‐21203; Thermo Fisher Scientific), for 4 h at room temperature under shielded light, and the nuclei were counterstained with DAPI. The specimens were imaged using an inverted microscope at ×200, and ImageJ software was used to measure the myofiber areas. Two central views of the transverse section with muscle damage were imaged, as previously described (Fujita et al., 2014). To evaluate the initial process of muscle regeneration, the number of muscle satellite cells was examined using Pax7 staining. The nuclei of newly regenerated muscle fibers were centrally located, whereas those of mature muscle fibers were peripherally located (Folker & Baylies, 2013). The rate of fibers with centrally located nuclei was measured from images of the dystrophin and DAPI (blue) co‐stained muscle sections, as previously described (Hettinger et al., 2021). Regenerated muscle fibers were also evaluated using type‐I MHC staining for identifying slow‐twitch fibers and type‐II MHC staining for fast‐twitch fibers; the ratio of slow/fast muscle fibers among all dystrophin‐stained muscle fibers was calculated.

### 2.9 Statistical analysis

Statistical analyses were performed using Student's *t*‐test. All analyses were performed using a statistical analysis software (JMP Pro 15; SAS Institute Inc., Cary, NC, USA). Statistical significance was set at *p* < 0.05. Data are expressed as mean ± standard deviation (SD).

## 3 RESULTS

### 3.1 VA promotes cell proliferation only in C2C12 cells and not in other adherent cells

**FIGURE 2:**
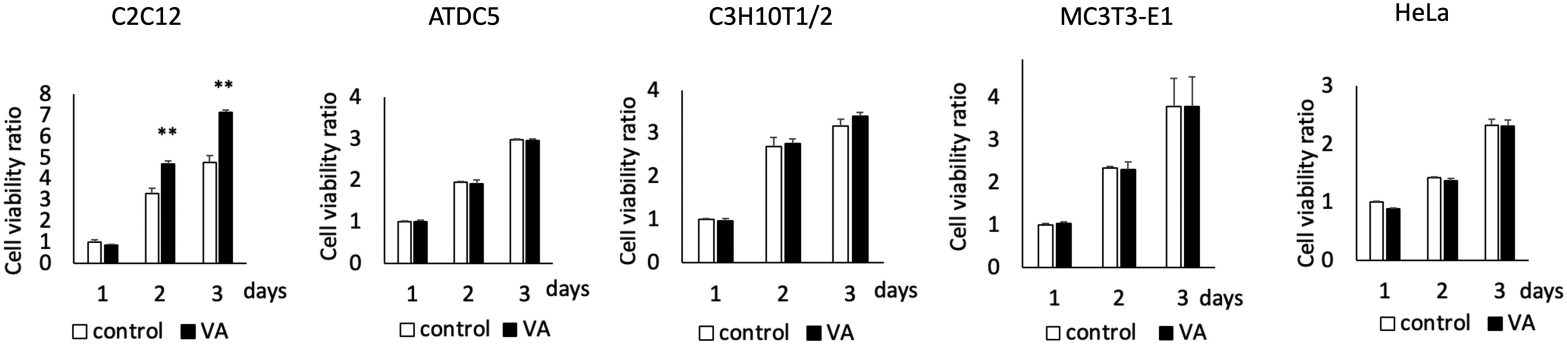
Quantification of cell proliferation in C2C12, ATDC5, C3H10T1/2, MC3T3‐E1, and HeLa cells using the WST/CCK‐8 assay. VA, vibration acceleration. *n* = 9 per time point per group. **Significant difference between groups, *p* < 0.01.

**FIGURE 3:**
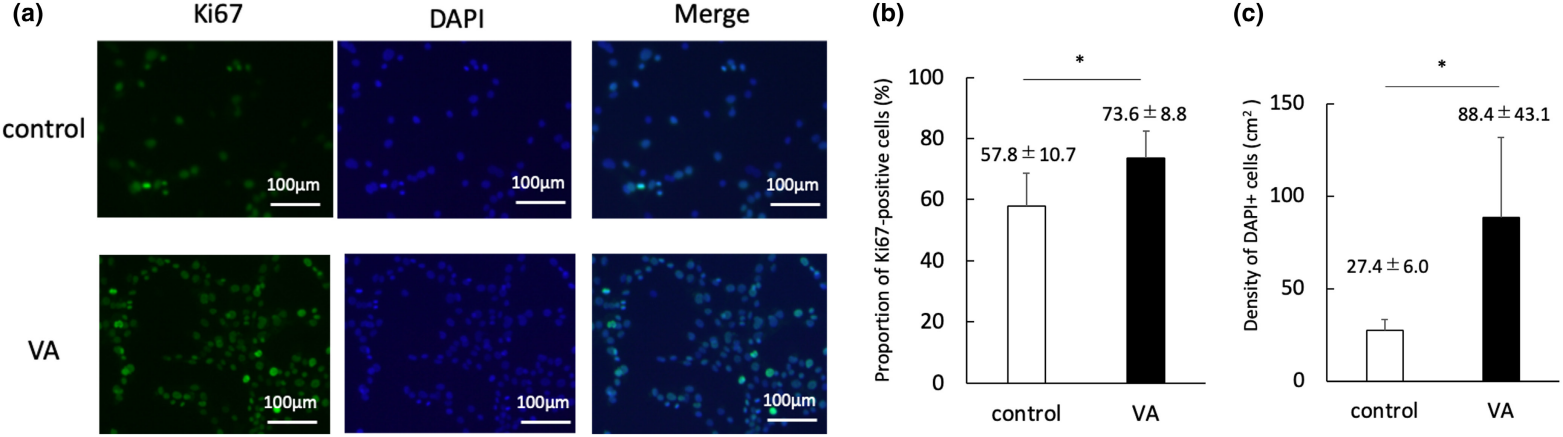
Evaluation of Ki67‐positive cells. (a) Representative images of immunostaining of C2C12 cells on Day 2 (Blue: nuclei stained with DAPI; Green: nuclei stained with Ki67). Bars indicate 100 μm. (b) The proportion of Ki67‐positive cells. (c) The density of DAPI positive cells. DAPI, 4′,6‐diamidino‐2‐phenylin‐dole; VA, vibration acceleration. *n* = 8 per group. *Significant difference between the groups, *p* < 0.05.

C2C12 and other adherent cells were exposed to VA at 30‐Low for 10 min/day, and WST/CCK‐8 assay revealed that VA enhanced cell proliferation in C2C12 cells on Days 2 and 3, but not in ATDC5, C3H10T1/2, MC3T3‐E1, and HeLa cells (Figure 2). As shown in Figure 3, the proportion of Ki67‐positive cells was significantly higher in the VA group (73.6%) compared to the control group (57.8%) on Day 2 (*p* < 0.05). The density of cells that were DAPI positive was 88.4 × 10^3^ cells/cm^2^ in the VA group, which was significantly higher than that in the control group (27.4 × 10^3^ cells/cm^2^).

### 3.2 VA increases C2C12 cell proliferation in a time‐dependent manner

**FIGURE 4:**
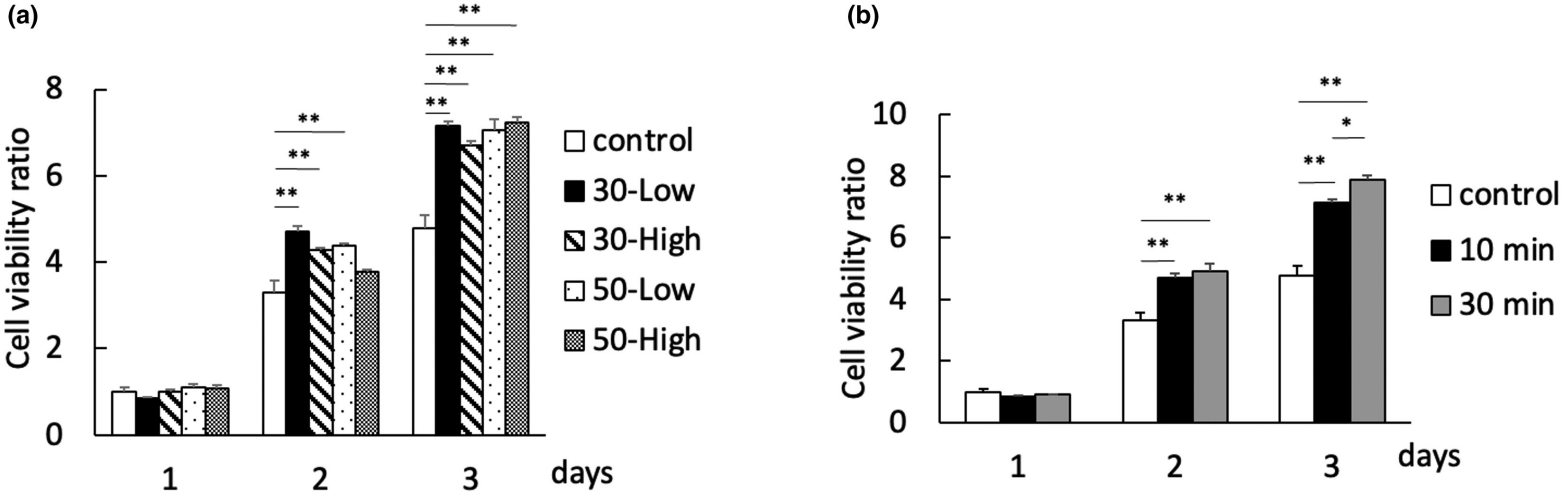
Differences of C2C12 proliferation by the WST/CCK‐8 assay at (a) various amplitudes for 10 min, and (b) various durations at 30‐Low. 30‐Low: 30 Hz/low amplitude, 30‐High: 30 Hz/high amplitude, 50‐Low: 50 Hz/low amplitude, 50‐High:50 Hz/high amplitude. *n* = 9 per time point per group. Significant difference between groups: *p < 0.05 and **p < 0.01.

Several different VA conditions (30‐Low, 30‐High, 50‐Low, and 50‐High, for 10 min/day) were used to stimulate C2C12 cells, without significant difference observed regarding the effect on cell proliferation (Figure 4a). However, when two different stimulation durations were compared (10 min/day versus 30 min/day at 30‐Low), 30‐min VA was found to promote cell proliferation more significantly than 10‐min VA (Figure 4b).

### 3.3 VA promotes C2C12 migration

**FIGURE 5:**
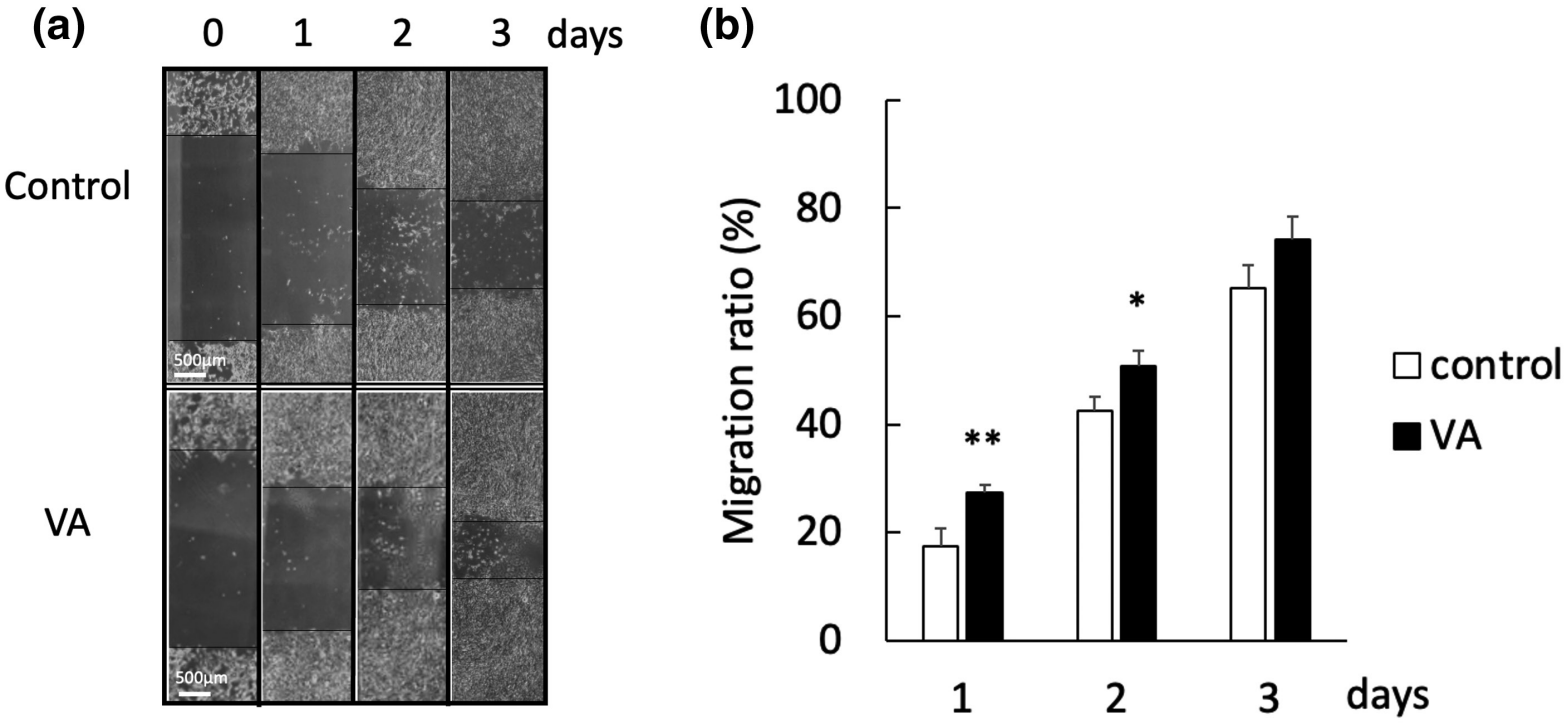
(a) Representative images showing migration of C2C12 cells on Days 0–3 after scratching, with or without VA. Bars indicate 500 μm. (b) Cell migration ratio on Days 1–3. VA, vibration acceleration. *n* = 15 per time point per group. Significant difference between groups: *p < 0.05, **p < 0.01.

Using the wound healing assay, the migration ratio was significantly higher in the VA group than that in the control group on Days 2 and 3 (Figure 5).

### 3.4 VA promotes myotube maturation but not gene expression related to differentiation

**FIGURE 6:**
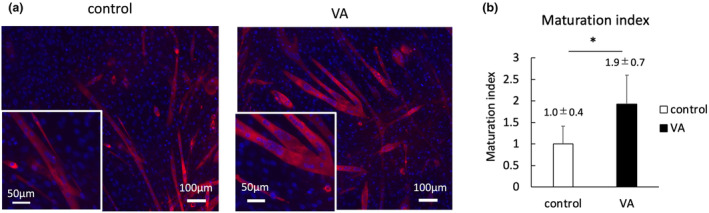
(a) Representative images of immunostaining of C2C12 myotubes on Day 11. Myosin heavy chain (MHC) and DAPI signals are stained red and blue, respectively. Bars indicate 100 μm and 50 μm. (b) Quantification of maturation index. DAPI; 4′,6‐diamidino‐2‐phenylin‐dole; VA, vibration acceleration. *n* = 10 per group. *Significant difference between the groups, *p* < 0.05.

**FIGURE 7:**
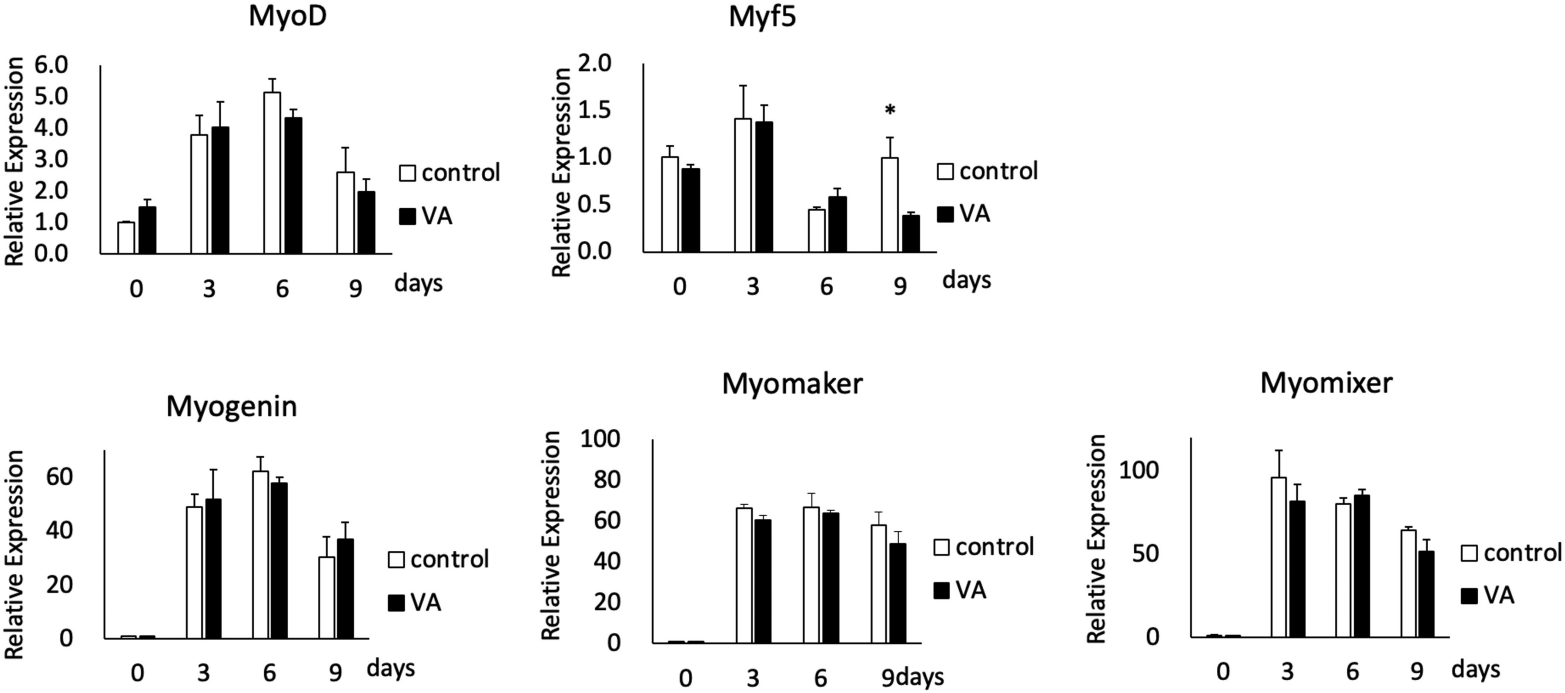
Relative expression of myogenic genes on Days 0, 3, 6, and 9. VA, vibration acceleration. *n* = 9 per time point per group. *Significant difference between groups, *p* < 0.05.

The maturation index was 1.9 fold higher in the VA group compared to the control group (Figure 6) (*p* < 0.05). In contrast, VA did not affect the expression of myogenic genes, such as *MyoD*, *Myf5*, *Myogenin*, *Myomaker*, and *Myomixer* (Figure 7).

### 3.5 VA promoted the muscle regeneration process

**FIGURE 8:**
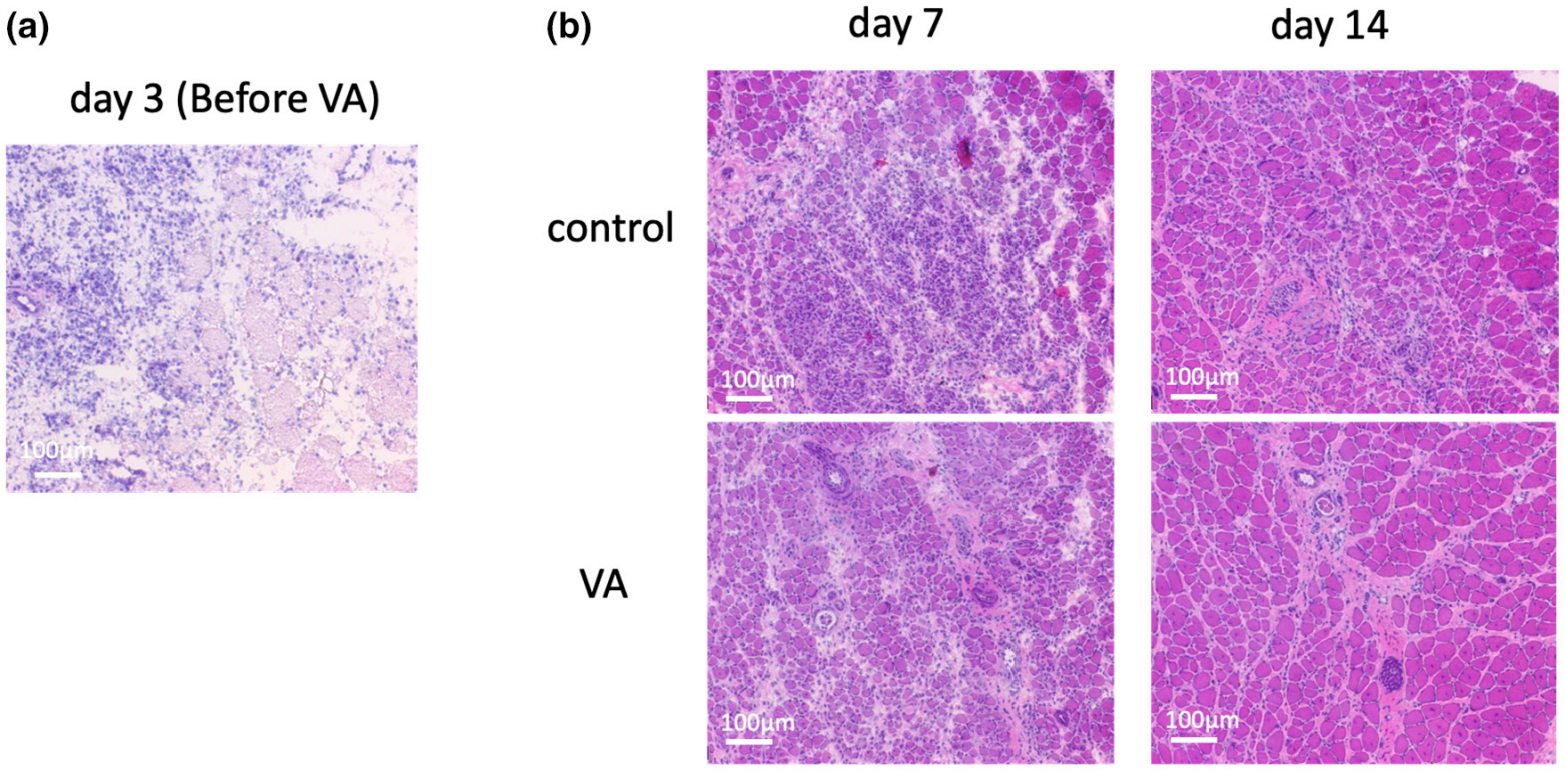
(a) Representative image of the soleus muscle on Day 3 of cardiotoxin injection, prior to vibration stimulation. (b) Representative images of regenerating muscle stained with hematoxylin and eosin on Days 7 and 14. Bars indicate 100 μm. VA, vibration acceleration.

At Day 3 after cardiotoxin injection, prior to vibration stimulation, soleus muscle fibers were destroyed and highly infiltrated with inflammatory cells, with few centrally nucleated muscle fibers and little muscle repair (Figure 8a). On Day 7, both groups appeared to have inflammatory cells and regenerating muscle fibers. However, on Day 14, the VA group appeared to have less inflammatory cell infiltration than did the control group (Figure 8b).

### 3.6 VA promotes proliferation of satellite cells in the early phase of muscle regeneration

**FIGURE 9:**
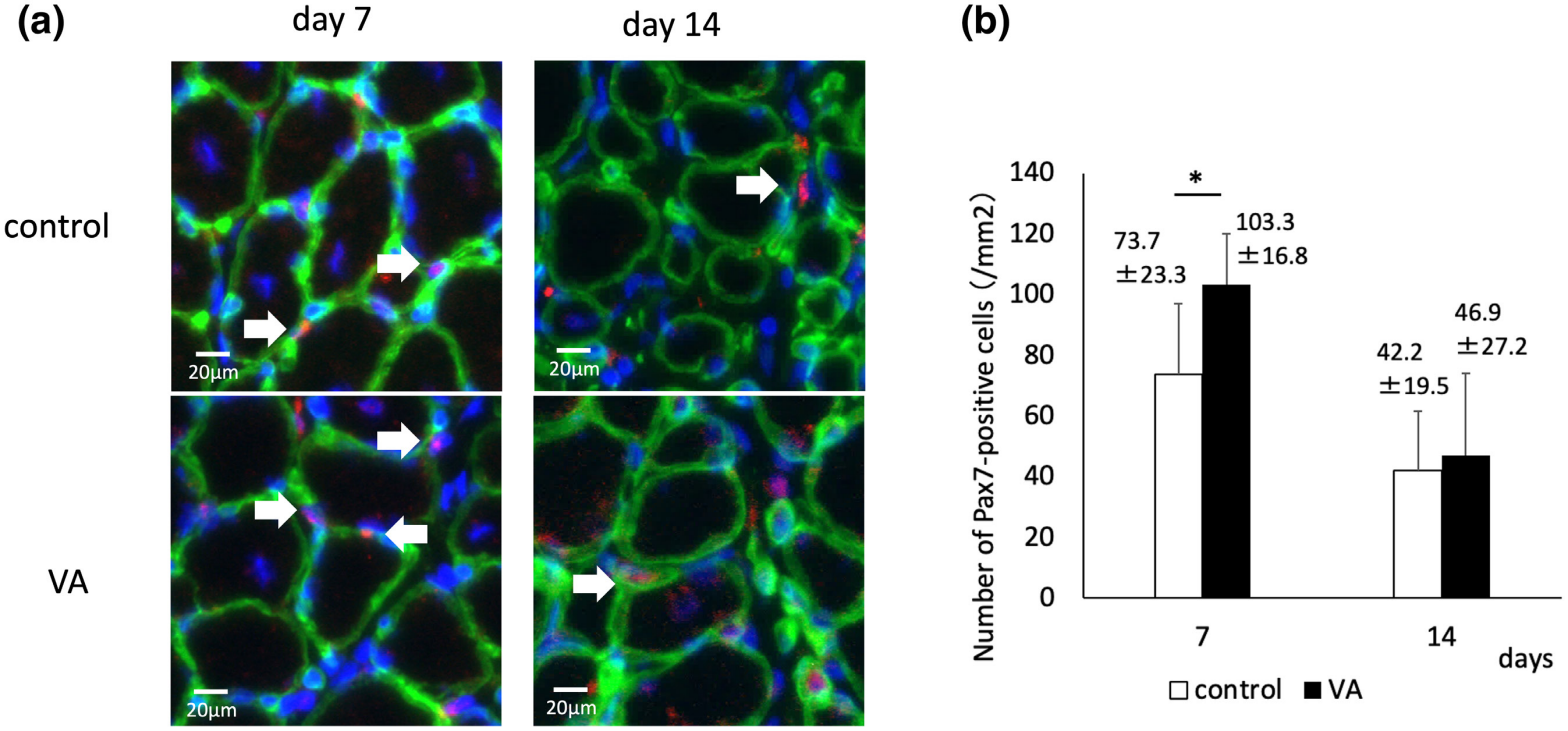
(a) Representative images of regenerated muscle fibers stained with Pax7 (red, arrow), laminin (green), and nuclei (blue); arrows stained with Pax7 indicate muscle satellite cells. Bars indicate 20 μm. (b) Number of Pax7‐positive cells is counted in 10 fields per two sections in six rats per time point per group. VA, vibration acceleration. *Significant difference between the groups, *p* < 0.05.

The number of muscle satellite cells was significantly higher in the VA group (103.3/mm^2^) than in the control group (73.7/mm^2^) on Day 7. However, there was no significant difference on Day 14 (Figure 9b).

### 3.7 VA promoted muscle fiber maturation

**FIGURE 10:**
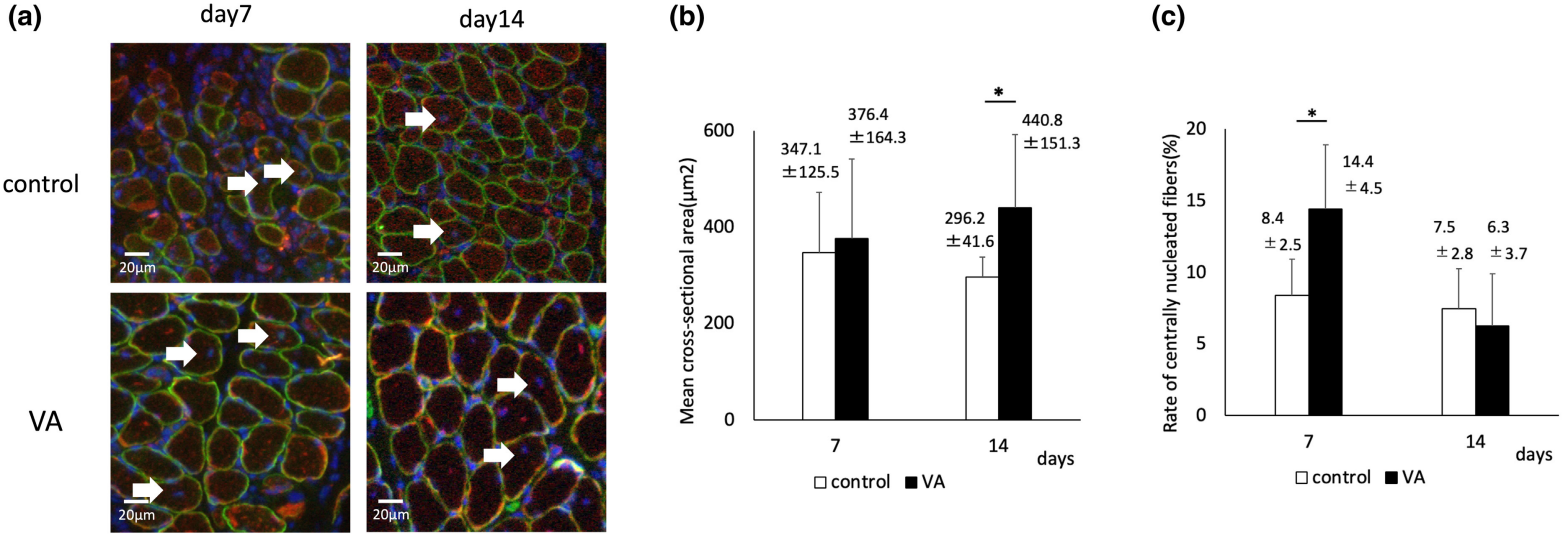
(a) Representative images of immunofluorescent staining for dystrophin (green), desmin (red), and nuclei (blue) indicate regenerated muscle fibers. White arrow indicates centrally located nuclei. Bars indicate 20 μm. The mean cross‐sectional area of regenerated muscle (b) and rate of centrally nucleated fibers (c) are measured in 10 fields per two sections in six rats per time point per group. VA, vibration acceleration. *Significant difference between the groups, *p* < 0.05.

**FIGURE 11:**
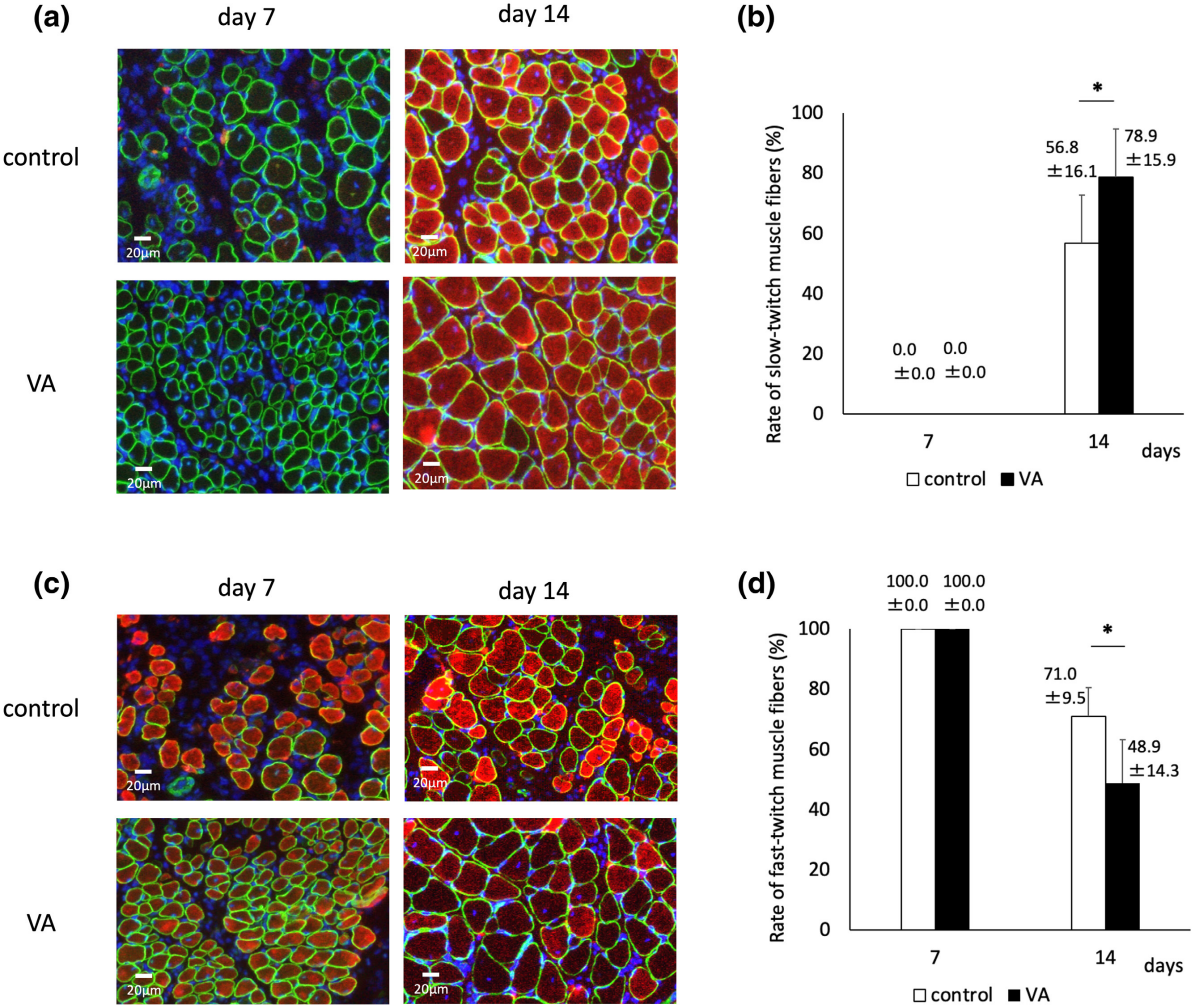
(a, c) Representative images of immunohistochemistry of muscle stained with dystrophin (green), nuclei (blue), (a) type‐I MHC (red) indicating slow‐twitch muscle, and (c) type‐II MHC (red) indicating fast‐twitch muscle. Rate of (b) slow‐twitch and (d) fast‐twitch muscle fibers are counted in 10 fields per two sections in six rats per time point per group. VA, vibration acceleration. *Significant difference between the groups, *p* < 0.05.

The mean cross‐sectional area of the regenerated muscle was not significantly different on Day 7 (Figure 10a). However, on Day 14, it was significantly larger in the VA group (440.8 μm^2^) compared to the control group (296.2 μm^2^) (Figure 10b). The rate of centrally nucleated fibers indicative of regenerating muscle was significantly higher in the VA group (14.4%) than that in the control group (8.4%) on Day 7. However, there was no significant difference on Day 14 (6.3% and 7.5%, respectively) (Figure 10c). On Day 7, almost all muscle fibers in both groups were fast‐twitch muscles. However, on Day 14, the VA group had significantly more slow‐twitch muscle fibers (78.9%) than did the control group (56.8%), as well as significantly fewer fast‐twitch muscle fibers (Figure 11). These results indicated that muscle regeneration was accelerated and further matured, and the transformation from fast‐ to slow‐twitch muscle fibers was promoted in the VA group.

## 4 DISCUSSION

In the present study, the in vitro and in vivo results indicated that VA may promote the proliferation and migration of satellite cells and accelerate the process of muscle regeneration in the drug‐induced muscle injury model. VA significantly promoted the proliferation and migration of C2C12 cells, a cell line derived from mouse skeletal satellite cells (Yaffe & Saxel, 1977), but did not affect the proliferation of other adherent cells (ATDC5, C3H10T1/2, MC3T3‐E1, and HeLa cells). There are two possible explanations for this observation. First, C2C12 cells may be more sensitive to VA compared to other cells. C2C12 had been reported to form more geometric shapes compared to endothelial cells or fibroblasts under mechanical stretch (Wang et al., 2014), indicating that cellular responses to mechanical stress may vary by cell types. Second, the stimulation conditions examined in this study may not be suitable for adherent cell lines other than C2C12 cells. The results of the present study also suggest that VA frequency in the range of 30–50 Hz and 10–30 min may be suitable for promoting C2C12 proliferation. In previous reports, 30 Hz/low‐amplitude VA for 10 min/day promoted endochondral ossification in vivo and chondrogenic differentiation in vitro (Yokoi et al., 2020), and since those conditions were considered most suitable for clinical application, the same conditions were used for the examination in this study.

The healing of injured skeletal muscle follows three phases: destruction, repair, and remodeling (Järvinen et al., 2005). When the skeletal muscle is injured, necrosis of myofibers and inflammatory cell reactions occur, followed by the activation and proliferation of satellite cells to initiate muscle regeneration. Proliferating satellite cells differentiate into myoblasts and join each other to form multinucleated myotubes. The newly formed multinucleated myotubes then fuse with the injured myofibers that have survived the initial trauma, and eventually, the regenerating parts of the myofibers take on their mature form (Hurme & Kalimo, 1992). Considering in vivo experiments, we used a rat muscle injury model created with a cardiotoxin, which can easily induce skeletal muscle injury and produce a comparable and reproducible response in age‐ and sex‐matched adult mice (Garry et al., 2016). In the present study, we targeted the soleus muscle, an anti‐gravity muscle predominantly comprising slow‐twitch fibers. Slow‐twitch muscle fibers are more sensitive to inactivity and, when damaged, take longer to repair and more likely to fail regeneration compared to damaged fast‐twitch muscle fibers (Zimowska et al., 2012, 2017). The soleus muscle is suitable for investigating changes from fast‐ to slow‐twitch muscle, which indicate maturation of repaired muscles. This would be clinically significant if methods for promoting slow‐twitch muscle regeneration are developed. The experiments revealed a significant increase in Pax7‐positive cells, a marker of satellite cells, in the VA group 7 days after muscle injury, and the mean cross‐sectional area of mature muscle fibers and the rate of slow‐twitch muscle fibers on Day 14 were significantly greater in the VA group. C2C12 proliferation was enhanced by VA in vitro, suggesting that VA may enhance the proliferation of satellite cells and promote regeneration of myofibers in vivo. WBV has been reported to increase myofiber size on Day 14, but not significantly on Day 7, in a mouse laceration injury model (Corbiere et al., 2018), which is similar to the results of the present study. It is known that myofiber type conversion from fast‐ to slow‐twitch muscle fibers occurs during muscle repair. The soleus muscle, a slow‐twitch‐dominant muscle, first regenerates as fast‐twitch muscle fibers after injury. Additionally, slow‐twitch muscle conversion is observed 4–5 days after injury (Matsuura et al., 2007). In the present study, VA not only increased size of the repaired muscle, but also promoted the conversion to slow‐twitch muscle, which may also promote functional recovery of injured muscle.

Regarding the effect of VA on C2C12 differentiation, myotube maturation was significantly accelerated on Day 11. However, qRT‐PCR did not show any promotion in the expression of genes involved in muscle differentiation. To our knowledge, two studies had reported that vibration enhanced the differentiation of C2C12 cells (Corbiere & Koh, 2020; Wang et al., 2010). Wang et al. (2010) found that vibrations at frequencies of 5, 8, and 10 Hz stimulated the expression of extracellular matrix proteins and myogenic regulatory factors in C2C12 cells. They also observed an increase in myotube formation, whereas no significant difference was found in cell viability between vibration‐treated cells and controls. Corbiere and Koh (2020) reported that loading 90 Hz local vibration for 30 min/day increased myotube size, but did not affect the expression of genes related to myogenesis or atrophy, similar to our results. Considering these reports, the possible reasons why VA did not promote the expression of genes related to muscle differentiation in this study could be that other genes may mediate the increase in differentiation or that the gene expression we evaluated may be altered at other time points. It is also possible that VA has a specific effect on muscle maturation. Furthermore, promotion of C2C12 proliferation was only observed in the present study, which may be due to the lack of standardized stimulation protocols and systematic investigations comparing different exposure conditions, leading to contradictory results.

A limitation of this study is that the molecular mechanisms underlying the effects of VA in C2C12 cells remain unknown. In previous reports on C2C12 cells, Da et al. (2020) reported that a cyclic strain promoted cell proliferation, which is consistent with our findings. They also indicated that mechanical stress could activate the PI3K signaling pathway and cyclin D gene expression. Hua et al. (2016) reported that the NF‐κB‐dependent miRNA profile was associated with cyclic mechanical stretch‐induced C2C12 cell proliferation. Other pathways reportedly involved in C2C12 proliferation, such as the AMPK and ERK pathways (Ha et al., 2019; Okamoto et al., 2014) might have played a role. Regarding genes involved in C2C12 differentiation, *mTOR* has been suggested to play a role in regulation of myoblast activation and differentiation during the muscle processes (Wang et al., 2018). Future studies are warranted to elucidate the specific molecular mechanisms underlying the effects of VA. Another limitation is that the function of the repaired muscles in rats, such as ankle torque, has not been investigated. Furthermore, the effect of VA should be examined in other injury models, such as contusion, and in other fast‐twitch‐dominant muscles. In addition, circadian clock may have influenced the results of this study. Recently, studies using clock‐disrupted mouse models have reported that muscle molecular clocks interacted with cell cycle components to facilitate myogenesis during injury (Kahn et al., 2023). Although satellite cells in skeletal muscles have been reported to have a functional molecular clock, their role in muscle regeneration is still largely unknown. Another limitation of this study is that the vibration stimulus was applied to the rat at 10:00 a.m. every day, without application at other times during the day.

In conclusion, we found that VA promoted C2C12 cell proliferation, migration, and maturation in vitro and accelerated the regeneration of rat cardiotoxin‐induced muscle in vivo. Our findings revealed that the effects of VA on muscle regeneration may be mediated by the direct effects on muscle cells.

## FUNDING INFORMATION

No funding was received for this work.

## CONFLICT OF INTEREST STATEMENT

The authors declare no conflict of interest.

## ETHICS STATEMENT

All animal experiments were approved by the Ethics Review Committee for Animal Experimentation of the Graduate School of Medicine, Osaka University (approval number: 27‐095‐000).

## Data Availability

The data that support the findings of this study are available from the corresponding author upon reasonable request.
